# Developing a Knowledge Translation Intervention to Improve the Detection and Management of Pediatric Dyslipidemias in British Columbia

**DOI:** 10.1016/j.cjcpc.2025.05.003

**Published:** 2025-05-27

**Authors:** Venessa K. Thorsen, Stephanie Glegg, Kevin C. Harris

**Affiliations:** a Children’s Heart Centre, BC Children’s Hospital, Vancouver, British Columbia, Canada; b Department of Medicine, the University of British Columbia, Vancouver, British Columbia, Canada; c BC Children’s Hospital Research Institute, Vancouver, British Columbia, Canada; d Department of Occupational Science and Occupational Therapy, University of British Columbia, Vancouver, British Columbia, Canada

**Keywords:** continuing medical education, dyslipidemia, familial hypercholesterolemia, implementation science, knowledge translation, pediatrics

## Abstract

**Background:**

Familial hypercholesterolemia (FH) is a common, underdiagnosed genetic condition associated with premature cardiovascular disease. Despite the availability of Canadian Cardiovascular Society (CCS)/Canadian Pediatric Cardiology Association (CPCA) guidelines, awareness and uptake among primary care providers remain limited. We developed and evaluated a continuing medical education (CME) course to improve adherence to pediatric dyslipidemia guidelines across British Columbia.

**Methods:**

We conducted a quasiexperimental pre-/post-knowledge translation study. The CME course was delivered in-person at BC Children’s Hospital and remotely to urban and rural family physicians and pediatricians. Pre-course and 1-month post-course surveys assessed self-reported confidence and adherence to CCS/CPCA recommendations.

**Results:**

Likelihood of screening pediatric patients for FH improved significantly after the course (*P* < 0.001), as did confidence in screening (*P* < 0.05) and diagnosing FH (*P* < 0.001). Screening based on risk factors increased significantly: at-risk race and ethnicity (+41%), cardiometabolic conditions (+51%), early-onset high cholesterol (+46%), family history of diabetes (+26%), and premature cardiovascular events in first-degree relatives (+57%). Adherence to diagnostic recommendations improved, including dietary and exercise counseling (+31%), dietician referral (+29%), family history assessment (+46%), and lipid specialist referral (+36%). Treatment adherence also increased: cascade screening (+14%), statin initiation (+23%), dietician referral (+24%), and lipid specialist referral (+36%). Most participants (93%) agreed or strongly agreed that they acquired new knowledge and found the CME to be the most effective format for guideline dissemination.

**Conclusions:**

The CME course effectively promoted CCS/CPCA guideline uptake and improved self-reported clinical practices. Expanding delivery to include trainees, nurses, and pharmacists may enhance impact and reach.

## Résumé

**Contexte:**

L’hypercholestérolémie familiale (HF), un trouble génétique courant mais sous-diagnostiqué, est associée à un risque de maladie cardiovasculaire prématurée. Malgré la publication de lignes directrices par la Société cardiovasculaire du Canada (SCC) et l’Association canadienne de cardiologie pédiatrique (ACCP), la sensibilisation à cette affection chez les fournisseurs de soins primaires et la capacité de dépistage de ces derniers demeurent limitées. Nous avons élaboré et évalué une activité de formation médicale continue (FMC) qui vise à améliorer l’application des lignes directrices en matière de prise en charge des dyslipidémies chez les enfants en Colombie-Britannique.

**Méthode:**

Nous avons mené une étude quasi expérimentale sur l’application de connaissances avant et après leur acquisition. L’activité de FMC a été offerte sur place, au BC Children’s Hospital, et en format virtuel à des médecins de famille et à des pédiatres exerçant en milieu urbain ou rural. Des questionnaires à remplir avant l’activité, puis 1 mois après celle-ci, ont permis aux médecins d’autoévaluer leur degré de confiance en matière de dépistage et le respect des recommandations formulées par la SCC et l’ACCP.

**Résultats:**

La probabilité de dépistage de l’HF chez les enfants était considérablement plus élevée après l’activité (p < 0,001), de même que la confiance des médecins à l’égard du dépistage (p < 0,05) de l’HF et de son diagnostic (p < 0,001). Le dépistage fondé sur les facteurs de risque a augmenté de manière significative, notamment en ce qui concerne les facteurs de risque liés à la race ou à l’origine ethnique (+41 %), à des affections cardiométaboliques (+51 %), à une hypercholestérolémie d’apparition précoce (+46 %), à des antécédents familiaux de diabète (+26 %) et aux manifestations cardiovasculaires prématurées chez des parents au premier degré (+57 %). L’application des recommandations en matière de diagnostic était plus prononcée, y compris les suivantes : conseils en matière d’alimentation et d’activité physique (+31 %), orientation vers une diététiste (+29 %), évaluation des antécédents familiaux (+46 %) et orientation vers un spécialiste des lipides (+36 %). L’application des recommandations en matière de traitement était également plus prononcée, y compris les suivantes : dépistage en cascade (+14 %), instauration d’un traitement par des statines (+23 %), orientation vers une diététiste (+24 %) et orientation vers un spécialiste des lipides (+36 %). La plupart des participants (93 %) étaient d’accord ou entièrement d’accord pour dire qu’ils avaient acquis de nouvelles connaissances; ils ont en outre estimé que le format de l’activité de FMC était le plus approprié pour assurer la diffusion des lignes directrices.

**Conclusions:**

L’activité de FMC a permis de promouvoir l’application des lignes directrices de la SCC et de l’ACCP, ce qui a eu une incidence positive sur la pratique clinique, selon l’autoévaluation des participants. Le fait d’offrir cette activité à un public élargi, notamment aux résidents, au personnel infirmier et aux pharmaciens pourrait accroître sa portée et ses répercussions positives.

Familial hypercholesterolemia (FH) is the most common inherited disorder of lipid metabolism and a major contributor to premature and accelerated atherosclerotic cardiovascular disease.^1^^,^^2^ Heterozygous FH affects approximately 1 in 250 individuals and leads to a 2- to 5-fold increase in low-density lipoprotein (LDL) cholesterol, substantially raising lifetime cardiovascular risk.^2^^,^^3^ Homozygous FH is far less common, affecting an estimated 1 in 160,000 to 300,000 individuals, but is more severe in presentation.^1^^,^^2^ It often manifests in childhood with a 4- to 6-fold elevation in LDL cholesterol and may rarely present with physical signs such as xanthelasmas, corneal arcus, and tendon xanthomas.^1^^,^^2^ Without intervention, nearly 85% of individuals with homozygous FH experience acute coronary syndrome by age 15 years.^4^^,^^5^ In heterozygotes, 7% are affected by age 30 years and 85% by age 60 years.^4^^,^^5^ Early initiation of statin therapy in childhood markedly reduces this risk.^3^^,^^5^ A 20-year longitudinal study published in *The New England Journal of Medicine* found that none of the participants who received early statin treatment experienced a coronary event, emphasizing the importance of early statin imitation in patients with FH.^3^^,^^5^

Elevated cholesterol can be detected easily by a full lipid profile, though surveys of family physicians and pediatricians demonstrate that screening is not done routinely in pediatric patients.^1^^,^^3^ Because of this lack of screening and the fact that dyslipidemias are clinically silent, approximately 90% of cases are not diagnosed during childhood currently.^1^ Numerous studies have demonstrated that early detection and treatment of dyslipidemias with statins has the potential to slow or even reverse early cardiac damage and to normalize cardiovascular risk in affected children.^1^

In August of 2022, the Canadian Cardiovascular Society (CCS) and the Canadian Pediatric Cardiology Association (CPCA) published updated clinical practice guidelines regarding pediatric dyslipidemias, such as FH.^3^ The new guidelines recommended universal screening for all children between the ages of 2 and 10 years and cascade screening for first-degree relatives of patients with diagnosed dyslipidemias, outline diagnostic criteria and thresholds, and point to the safety and efficacy of statin use in pediatric patients with FH.^3^ Similarly, the American Heart Association’s most recent guidelines recommend universal screening for children aged 9-11 years to improve early detection of lipid disorders, with screening potentially starting as early as age 2 years for children with a family history of early heart disease and other risk factors.^6^ However, recent national and provincial surveys have shown no meaningful change in screening rates despite the presence of these guidelines, underscoring the importance of effective knowledge translation (KT) initiatives to ensure that the guidelines are translated into practice and lead to improvements in early detection and care.^1^ The CCS, CPCA, and American Heart Association have all identified KT of guideline content to family physicians and pediatricians, as the most pressing future direction.^3^^,^^6^

The objective of this study was to evaluate an interactive KT intervention to improve the knowledge, awareness, and potential use of CCS/CPCA guideline recommendations by family doctors and pediatricians across urban, suburban, and rural/remote settings in British Columbia (BC).

## Methods

### KT theory

The intervention was designed to address common barriers faced by health care providers when adopting new clinical practices, drawing on principles from the Behaviour Change Wheel.^7^ Common barriers include time constraints, limited access to clinical guidelines, and physician motivation.^7^^,^^8^ To address time constraints, the intervention offered multiple synchronous sessions with recordings for later viewing, along with an asynchronous version to provide flexible participation. This approach increased opportunities for engagement by making learning accessible without additional time burdens. A 1-page summary of key dyslipidemia screening and management guidelines was also provided, offering a concise reference to support clinical decision-making. The relevant guidelines were embedded directly into the intervention and distributed to participants after the course, enhancing accessibility and supporting the application of the guidelines in practice. Physician motivation was addressed through continuing medical education (CME) accreditation, which aligned with physicians' professional development goals. By offering a credential that contributes to continued education, the intervention appealed to intrinsic motivations for growth and learning.^9^ The credibility of the content was strengthened by involving pediatric cardiologists in the delivery of the material. This helped to foster a sense of trust and collaboration, which is essential for effective learning.^7^^,^^9^ Reflection activities throughout the course also supported knowledge retention and long-term behavior change.^10^^,^^11^ By encouraging participants to consider how the material could be applied to their practice, these activities helped internalize the content and promote sustained engagement.^10^^,^^11^

### KT intervention design

The intervention was a CME course accredited by the College of Family Physicians of Canada (CFPC) and the Royal College of Physicians and Surgeons of Canada, designed for family physicians and pediatricians across BC. Delivered through both virtual and in-person formats, the course focused on 3 pediatric clinical cases related to dyslipidemia, in line with the 2022 CCS/CPCA guidelines (Supplemental Appendix S1). The course included a survey of demographics (Supplemental Appendix S2), live, interactive question-and-answer sessions and polling, as well as case-based learning to engage participants and facilitate practical application of the material. Three short, prerecorded video lectures provided the guidelines for primary dyslipidemia detection and management in pediatric patients, followed by a live question-and-answer session led by pediatric cardiologists. On completion, participants received a copy of the guidelines, 1-page summary of key points, and flow chart for clinical decision-making via email (Supplemental Appendix S3, Supplemental Figs. S1 and S2). A 1-month follow-up was selected to assess short-term knowledge retention and early shifts in confidence and intent to change practice. Future work will explore longer-term impacts by examining population trends in pediatric lipid screening, diagnosis, and treatment across BC.

### Inclusion/exclusion criteria

Licensed family physicians and pediatricians in BC, including those in academic and community-based practices, were eligible to participate in data collection. Physicians from other provinces, specialties outside of family medicine or pediatrics, and medical students were excluded from the study.

### Participant recruitment

Participants were recruited primarily through email lists compiled by physician associations, such as BC Family Doctors, the BC College of Family Physicians, the University of British Columbia (UBC) Department of Pediatrics, the BC Pediatric Society, Doctors of BC, Divisions of Family Practice, and the Rural Coordination Centre of BC (RCCbc). Visits to medical clinics, phone and fax contact/follow-up with clinics, and snowball sampling of first- and second-year UBC medical students’ family practice preceptors and their colleagues supplemented email recruitment. Recruitment was also supported through an opt-in function on a concurrent provincial survey of practices, attitudes, and barriers to lipid screening and management, administered by members of our study team.

### Ethics statement

This study was approved by the joint UBC-Children's and Women's Behavioural Research Ethics Board. Informed consent was obtained from all participating physicians before data collection.

### Outcomes of interest

The primary outcomes of this study were (1) self-reported adherence to guideline recommendations before and 1 month after the CME course. The secondary outcomes included (1) self-reported confidence in screening, diagnosing, and treating genetic pediatric dyslipidemias before and 1 month after the CME course; (2) intentions to change practice; and (3) physicians’ satisfaction with the CME course.

### Implementation process evaluation

To evaluate the success of the intervention, we assessed several key implementation process metrics, including reach, dose, fidelity, participant satisfaction, and engagement with reflection activities.^7^^,^^12^, ^13^, ^14^ Reach was measured by tracking participant registration and attendance, ensuring that the target audience of pediatricians and family physicians was effectively engaged. Dose was evaluated by monitoring participation in interactive components, such as question-and-answer sessions and polling activities. Fidelity was assessed through feedback from facilitators and participants to ensure adherence to the original design and content. Satisfaction was measured through postsession surveys, evaluating participant perceptions of the course's relevance and clarity. Engagement with reflection activities was also tracked to gauge the impact on knowledge retention and potential practice changes. These metrics provided a comprehensive evaluation of the intervention’s effectiveness in reaching and engaging participants, as well as its potential to influence behavior change in clinical practice.^13^^,^^14^

### Data collection

A 1-time demographics survey and 1-month follow-up surveys were administered via REDCap (Research Electronic Data Capture), a secure, web-based application designed to support data capture for research studies.^15^ Live polling during the CME sessions was facilitated using “PollEverywhere.” The demographics survey collected participant characteristics, including age, sex, years in practice, medical specialty, community size, practice environment, health authority affiliation, and whether they identified as the primary provider for their patients (Supplemental Appendix S2). The intervention incorporated live polling and follow-up surveys, using a combination of 5-point Likert scales, forced-choice, and open-text questions. These tools assessed various aspects of provider confidence, adherence to existing CCS guidelines for pediatric dyslipidemias, and engagement with clinical cases. Participants were asked to self-report their likelihood of screening, diagnosing, and treating hypothetical patients, as well as their course satisfaction and intention to change practice.

### Analyses

Descriptive statistics including median [interquartile range] (continuous variables), and frequencies and percentages (binary/categorical variables) were used to summarizer applicable demographic variables. Distribution of continuous variables was assessed visually for normality by inspecting histogram plots. Wilcoxon rank-sum tests (nonparametric, independent) were used to analyze potential differences in median age and years of practice between family physicians and pediatricians. The frequencies of self-reported CCS/CPCA-recommended strategies used before and 1 month after the course were compared, along with physicians' confidence in managing pediatric dyslipidemias. Bivariate analyses for 2 categorical variables were assessed using χ^2^ tests (parametric and independent) where assumptions were met and using the Fisher exact test (nonparametric and independent) where they were not met. A 2-sided *P* value of <0.05 was considered significant. All analyses were conducted in R (v. 3.6.2) using R Studio (v.1.1.456) (R Foundation for Statistical Computing, Vienna, Austria).^16^

## Results

### Participant demographics

**Figure 1:**
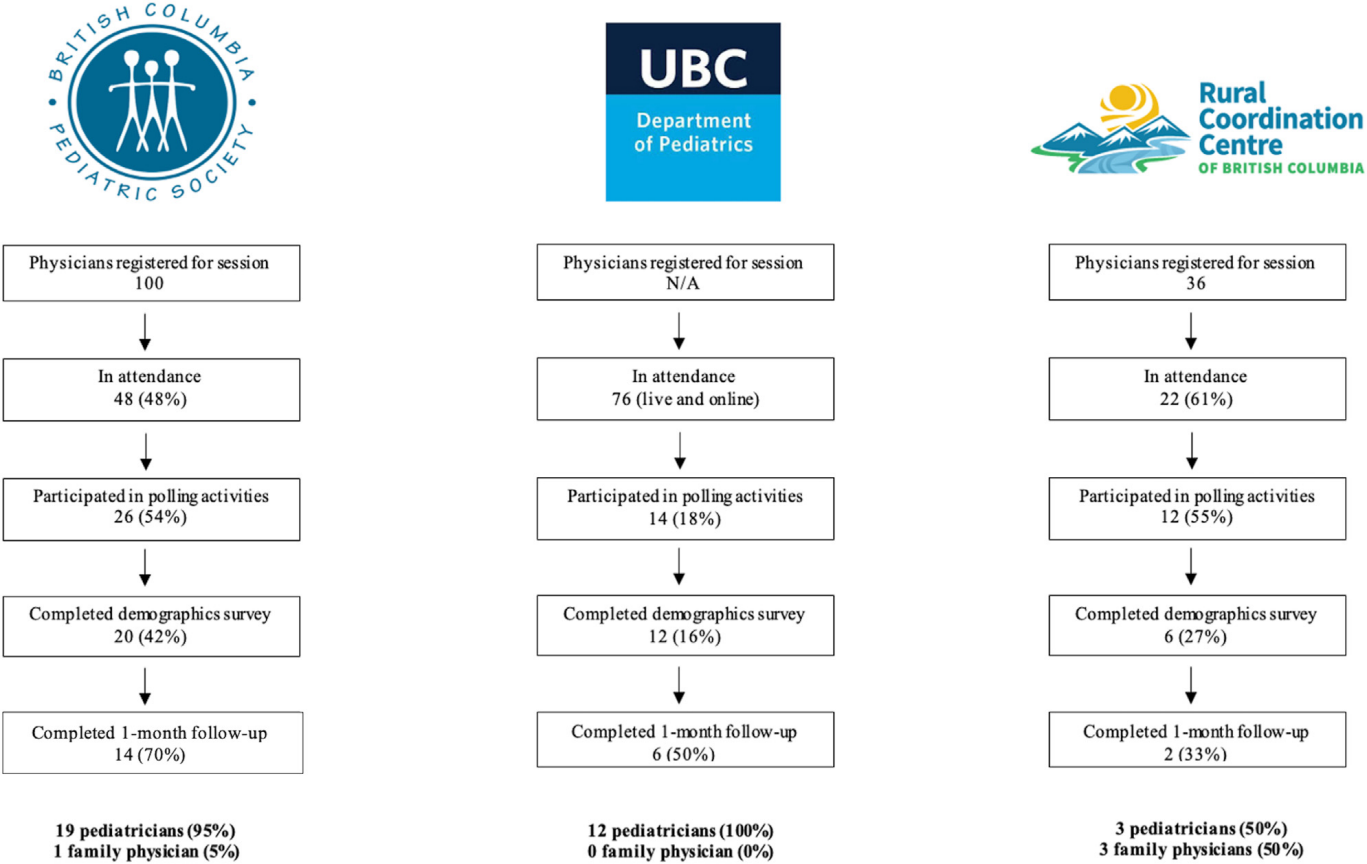
Flow chart diagram of study recruitment and intervention retention for British Columbia Pediatric Society and University of British Columbia (UBC) Pediatrics Grand Rounds Continuing Medical Education sessions. N/A, not applicable.

Over 3 live CME sessions held between December 2023 and June 2024, and 146 physicians attended, with 52 (36%) participating in data collection through REDCap and Poll Everywhere. Thirty-eight participants (26%) consented to provide demographic data, and no participants opted for the asynchronous module. Of the 52 who engaged in polling activities at the first study time point, 21 (40% retention) participated at the 1-month follow-up (Fig. 1).

Thirty-eight physicians consented to provide demographic data. Twenty-eight of 38 (74%) were female, with a median age of 46.5 years (Table 1), whereas 61% (23 of 38) considered themselves to be the primary care giver to most/all their patients. Twenty-one (55%) physicians practiced independently, whereas 17 (45%) were members of a care team (Table 1). Fifty percent of respondents (19 of 38) were community-based pediatricians (including subspecialty), and the predominant practice environments were private clinics (17 respondents, 45%) and inpatient hospital (14 respondents, 37%) (Table 1). The sample represented all 5 geographic health authorities in BC (Table 1).

**Table 1:** Continuing medical education course participant demographics

Demographic	Value, n (%) or median [IQR]
Age (y)	46.5 [19.75]
Years of practice	18 [19]
Medical specialty	
Family physician	4 (11)
Pediatrician	34 (89)
Sex	
Male	10 (26)
Female	28 (74)
Primary care provider	
Yes	23 (61)
No	15 (39)
Practice environment	
Independent	21 (55)
Member of a care team	17 (45)
Community size	
Urban	27 (71)
Suburban	7 (18)
Rural/remote	4 (11)
Primary practice setting∗	
Inpatient hospital	14 (37)
Hospital outpatient clinic	8 (21)
Private office	17 (45)
Community setting	3 (8)
Other setting	2 (5)
Specialty	
Community-based family physician	4 (11)
Community-based pediatrician	16 (42)
Community-based subspecialty pediatrician	3 (8)
Academic-based pediatrician	2 (5)
Academic-based subspecialty pediatrician	11 (29)
Other	2 (5)
Regional health authority	
Vancouver Coastal Health	19 (51)
Interior Health	9 (24)
Island Health	4 (11)
Fraser Health	4 (11)
Northern Health	1 (3)

IQR, interquartile range.

Asterisks (∗) denote questions where multiple responses were permitted.

### Practitioner experience

**Figure 2:**
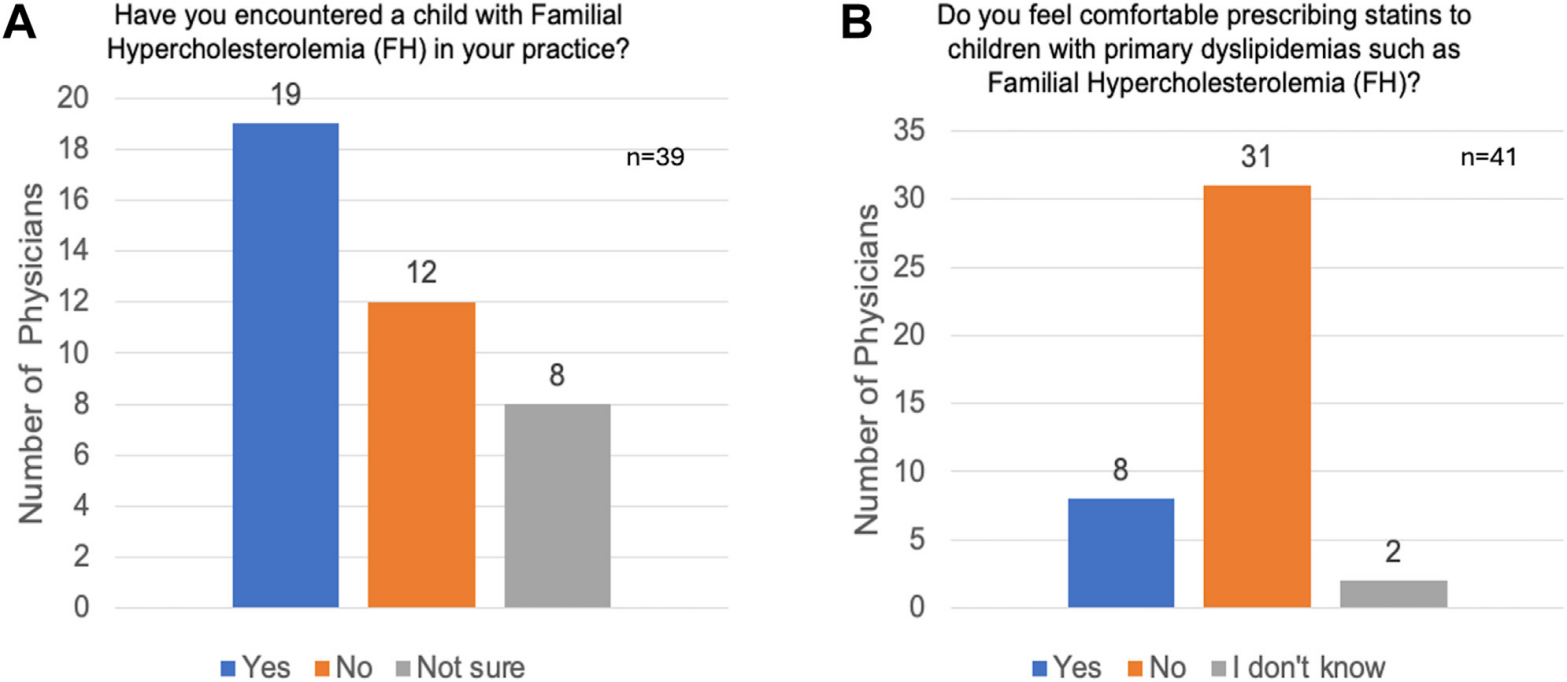
Number of physicians who believed that they had encountered a child with FH in their practice (**A**) and number of physicians who felt comfortable prescribing statins to children with primary dyslipidemias before a continuing medical education course (**B**).

Before the course, 19 of 39 physicians (49%) reported having encountered a child with FH before; 31 of 41 physicians (76%) did not have experience with or were not comfortable prescribing statins to children with genetic dyslipidemias (Fig. 2).

### Universal pediatric FH screening practices increased after intervention

**Figure 3:**
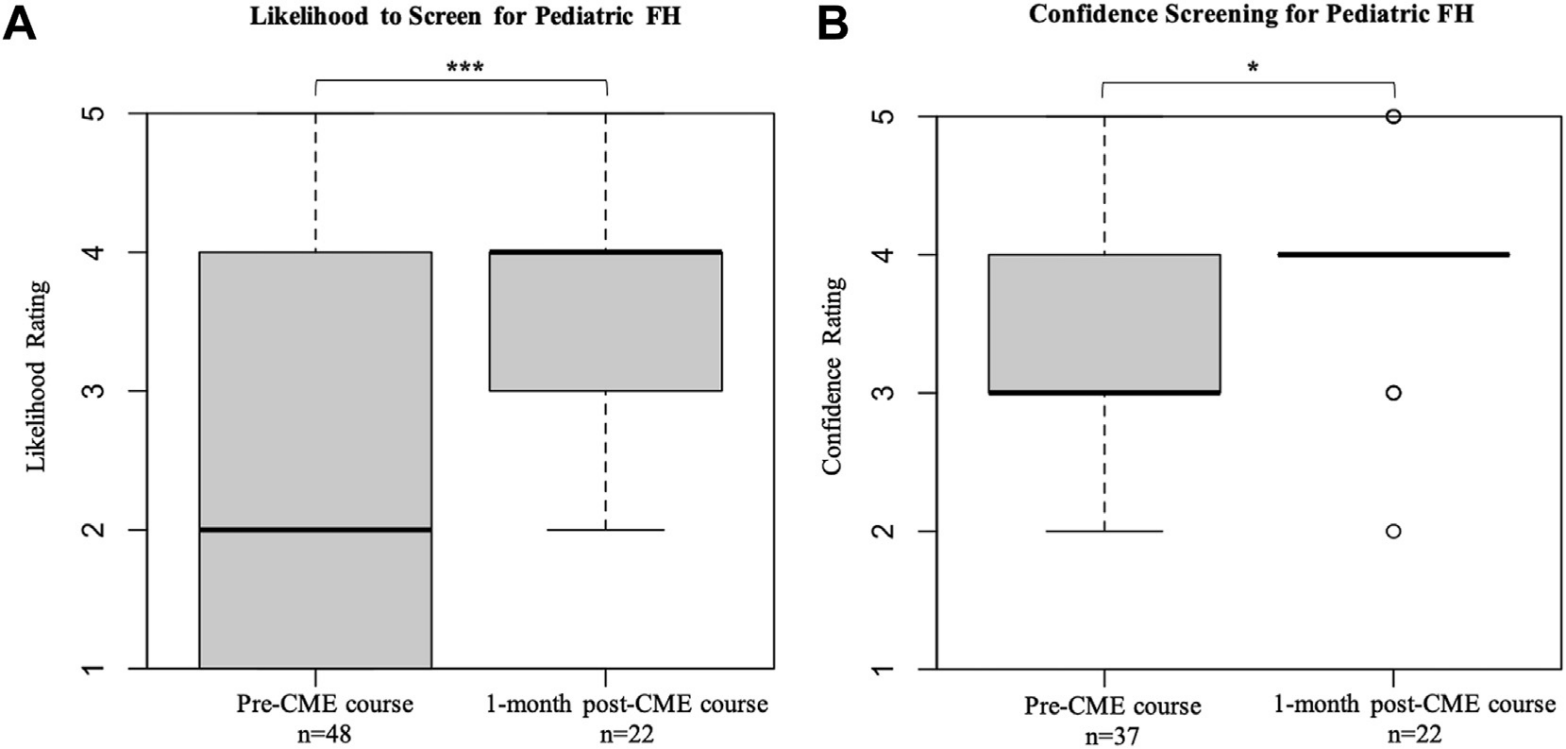
Physician self-reported likelihood to screen a healthy pediatric patient for FH before and 1-month after participating in a CME course (*P* < 0.001) (**A**) and physician self-reported confidence screening for pediatric FH before and after a CME course (*P* < 0.05) (**B**). CME, continuing medical education; FH, familial hypercholesterolemia.

Median ratings for likelihood to screen a healthy pediatric patient for FH improved significantly from preintervention (median rating = 2, unlikely) to postintervention (median rating = 4, likely) (*P* < 0.001) (Fig. 3A).

### Screening strategies used before CME

Before the course, factors that physicians reported considering when deciding whether or not to screen pediatric patients for FH and other dyslipidemias were comorbidities, including chronic kidney disease, hypertension, and Kawasaki disease (26%); medications, such as antipsychotic drugs, risperidone, or chemotherapy (26%); physical examination findings, such as xanthomas and acanthosis nigricans (22%); and situational factors, such as age, convenience (ie, if blood work is being taken already) (17%), and parental concern (9%).

### Confidence screening for pediatric FH improved after intervention

Median confidence ratings in screening pediatric patients for FH significantly improved from preintervention (median rating = 3, neutral) to postintervention (median rating = 4, somewhat confident) (*P* < 0.05) (Fig. 3B).

### Screening rates increased after intervention

Physicians reported increases in screening rates, particularly in children with additional cardiovascular risk factors after the CME interventions (see Table 2). Particular risk factors for which a significant increase in screening rates was reported included at-risk race and ethnicity (+41%), chronic cardiometabolic conditions (+51%), family history of early-onset high cholesterol (+46%), family history of diabetes (+26%), and first-degree relatives with premature heart attack or stroke (+57%) (*P* < 0.05). However, no significant change in screening behaviors was reported in the context of working with children who were overweight or obese (*P* = 1).

**Table 2:** Self-reported pediatric FH screening rates based on guideline risk factors before and after the CME course

	Before CME course (n = 39), n (%)	1 mo after CME course (n = 22), n (%)	Margin of change	*P* value
Overweight/obese				
Yes	36 (92)	20 (91)	–1%	1
No	3 (8)	2 (9)		
At-risk race and ethnicity				
Yes	9 (23)	14 (64)	+41%	0.003
No	30 (77)	8 (36)		
Chronic cardiometabolic condition (eg, hypertension and diabetes)				
Yes	17 (44)	21 (95)	+51%	<0.001
No	22 (56)	1 (5)		
Family history of early-onset high cholesterol				
Yes	21 (54)	22 (100)	+46%	0.0001
No	18 (46)	0 (0)		
Family history of diabetes				
Yes	8 (42)	15 (68)	+26%	0.00003
No	31 (58)	7 (32)		
First-degree relative with premature heart attack or stroke				
Yes	15 (38)	21 (95)	+57%	<0.00001
No	24 (62)	1 (5)		

CME, continuing medical education; FH, familial hypercholesterolemia.

### Confidence diagnosing pediatric FH improved after intervention

**Figure 4:**
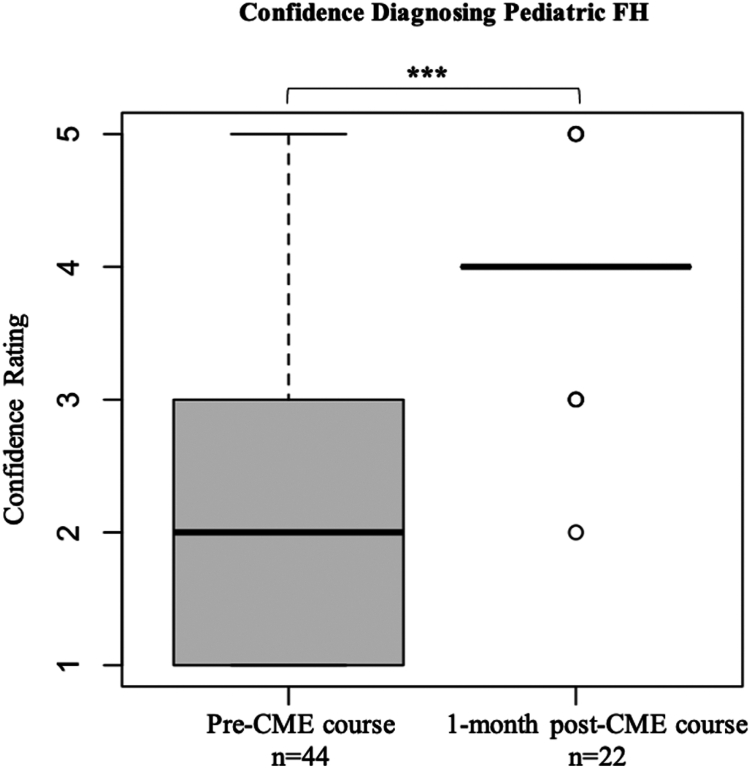
Physician self-reported confidence diagnosing pediatric FH before and after a CME course (*P* < 0.001). CME, continuing medical education; FH, familial hypercholesterolemia.

Median confidence ratings for diagnosing pediatric dyslipidemias, such as FH, improved significantly from preintervention (median rating = 2, somewhat unconfident) to postintervention (median rating = 4, somewhat confident) (*P* < 0.001) (Fig. 4). Although correct FH diagnoses based on 2 abnormal lipid profiles improved by 21%, this increase was also not significant (*P* > 0.05).

### Adherence to management recommendations increased after intervention

Self-reported adherence to several CCS/CPCA guideline recommendations for managing a hypothetical child with an LDL cholesterol profile diagnostic of FH improved significantly from pre-course to 1 month post-course (see Table 3).

**Table 3:** Self-reported use of diagnostic strategies for pediatric FH before and after the CME course

	Before CME course (n = 50), n (%)	1 mo after CME course (n = 22), n (%)	Margin of change	*P* value
Repeat cholesterol test in 3 months without other changes				
Yes	3 (6)	2 (9)	+3%	0.6379
No	47 (94)	20 (91)		
Provide ongoing dietary and exercise counseling				
Yes	23 (46)	17 (77)	+31%	0.0201
No	27 (54)	5 (23)		
Start statin				
Yes	22 (44)	13 (59)	+15%	0.3083
No	28 (56)	9 (41)		
Refer to dietician				
Yes	22 (44)	16 (73)	+29%	0.0394
No	28 (56)	6 (27)		
Offer genetic testing				
Yes	12 (24)	10 (45)	+21%	0.0961
No	38 (76)	12 (55)		
Assess family history				
Yes	23 (46)	19 (86)	+40%	0.0016
No	27 (54)	3 (14)		
Conduct physician examination				
Yes	27 (54)	15 (68)	+14%	0.3074
No	23 (46)	7 (32)		
Refer to lipid specialist				
Yes	23 (46)	18 (82)	+36%	0.0051
No	27 (54)	4 (18)		
Diagnose patient with familial hypercholesterolemia				
Yes	10 (20)	9 (41)	+21%	0.0839
No	40 (80)	13 (59)		

CME, continuing medical education; FH, familial hypercholesterolemia.

### Patient management before CME

Given a scenario of a child with a cholesterol profile consistent with FH, physicians reported pursuing referrals to endocrinologists, geneticists, obesity programs, and cardiology services (54%-67%), and cascade screening of siblings and parents (33%). Additional management avenues employed by physicians included monitoring of blood pressure and glycemic controls (23%), and supplemental treatments such as high fiber diets and plant sterols (23%). Few would consider diagnosing FH (7%) or treating with statins (11%) before the KT intervention, despite support for this approach in the guidelines.

### Adherence to treatment recommendations increased after intervention

Self-reported adherence to CCS/CPCA guideline recommendations for treating confirmed cases of FH and other pediatric dyslipidemias improved from pre-CME to 1 month post-CME. The improvements were as follows: provide ongoing dietary and exercise counseling (+6%), recommend cascade screening for dyslipidemias among first-degree relatives (+14%), start statin (+23%), refer to dieticians (+24%), and refer to lipid specialists (+36%). However, the only improvement that was statistically significant was referrals to lipid specialists (*P* = 0.0005) (Table 4).

**Table 4:** Self-reported use of treatment strategies for pediatric FH before and after the CME course

	Before CME course (n = 49), n (%)	1 mo after CME course (n = 22), n (%)	Margin of change	*P* value
Provide ongoing dietary and physical activity counseling				
Yes	35 (71)	17 (77)	+6%	0.774
No	14 (29)	5 (23)		
Recommend cascade screening for dyslipidemias among first-degree relatives				
Yes	29 (59)	16 (73)	+14%	0.3019
No	20 (41)	6 (27)		
Start statin				
Yes	22 (45)	15 (68)	+23%	0.0792
No	27 (55)	7 (32)		
Refer to dietician				
Yes	24 (49)	16 (73)	+24%	0.0747
No	25 (51)	6 (27)		
Refer to lipid specialist				
Yes	23 (47)	20 (91)	+44%	0.0005
No	26 (53)	2 (9)		

CME, continuing medical education; FH, familial hypercholesterolemia.

### Confidence treating pediatric FH improved modestly after intervention

**Figure 5:**
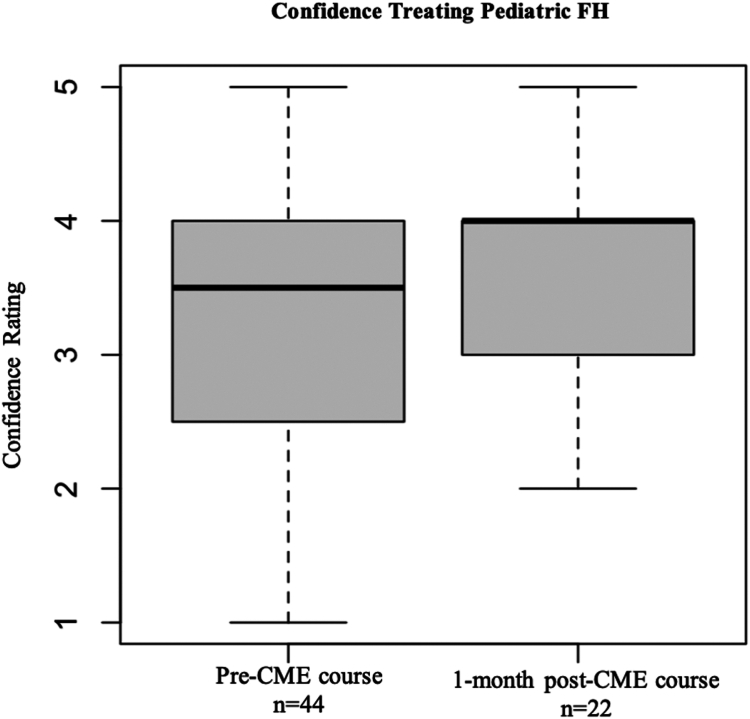
Physician self-reported confidence treating pediatric FH before and after a CME course. CME, continuing medical education; FH, familial hypercholesterolemia.

Median confidence ratings for treating pediatric dyslipidemias, such as FH, improved from preintervention (median rating = 3.5, between neutral and somewhat confident) to postintervention (median rating = 4, somewhat confident); however, this improvement was not statistically significant (*P* = 0.4062) (Fig. 5).

### Reflection and CME course evaluation

#### Reach, dose, and fidelity

A total of 146 physicians attended the CME course. Of these, 52 (36%) participated in interactive components, including question-and-answer sessions and polling activities, and 48 (33%) completed the final reflection and course evaluation. Fidelity was assessed through facilitator and participant feedback, which indicated adherence to the original course design and content.

#### Participant satisfaction

Median course satisfaction was high (median rating = 4, satisfied), with no significant difference between immediate post-course and 1-month post-course ratings (*P* = 0.2176).

Ninety-three percent (42 of 45) of participants agreed or strongly agreed that the course was the most effective presentation of the CCS and CPCA guidelines, whereas 7% (3 of 45) responded “neutral.” Participants identified course length, pacing, clarity, content, short video lectures, and interactive components as the most favorable aspects.

### Engagement with reflection activities and reported practice changes

Among those who completed the final reflection, 98% (47 of 48) reported learning new information. Key takeaways included the recommendation for universal screening of dyslipidemias (61%, n = 29), the prevalence of FH (14%, n = 7), FH diagnostic criteria (2%, n = 1), and statin therapy considerations (23%, n = 11).

Median agreement ratings for intention to change practice immediately after the course did not differ significantly from self-reported practice changes at 1 month (*P* = 0.2434). Immediately after the course, 71% (n = 34) of participants indicated intentions to implement universal screening, 4% (n = 2) planned to review FH diagnostic criteria, and 26% (n = 12) intended to initiate statin therapy. At 1 month, 93% (n = 19) reported screening most or all pediatric patients for FH, and 9% (n = 2) had prescribed statins to children with uncontrolled LDL cholesterol.

## Discussion

The success of our KT intervention in enhancing knowledge and adherence to CCS/CPCA guidelines for pediatric dyslipidemias, along with high participant satisfaction, demonstrates the effectiveness of our intervention for educating and creating intention to change practice among pediatricians and family physicians.^17^, ^18^, ^19^, ^20^ Based on population estimates, approximately 9800 children in BC have FH. Screening rates are known to be low, around 3%-5%, suggesting that approximately 9300 of these patients are likely undiagnosed.^2^^,^^3^ The results of our study suggest that a meaningful opportunity exists to reduce the gap in diagnosis and to improve treatment rates for pediatric dyslipidemias. Increases in reported utilization of guideline-congruent screening, diagnosis, and treatment strategies by participating family physicians and pediatricians suggest promise for enhanced detection and management of FH and other primary genetic dyslipidemias in BC.^2^^,^^3^ This finding lends support to the potential for KT interventions to enhance preventive cardiac care for children. Children with genetic dyslipidemias are at high risk of delayed diagnosis because of the clinical silence of these disorders in this population and the prolonged lag time between the onset of dyslipidemia and clinical cardiovascular events.^1^^,^^3^^,^^21^^,^^22^ Although our findings regarding the impact of a KT intervention are promising, scaling the intervention to impact screening rates at a population level is needed.

Resistance or inaction within the medical community, despite established recommendations from both American and Canadian medical societies, remains a significant challenge in addressing FH.^1^^,^^3^ Additional barriers may include limited awareness, competing clinical priorities, a lack of understanding among physicians regarding the necessity of addressing dyslipidemias like FH in childhood, and the long-term impact of early preventive care.^1^ Competing messaging and inconsistent guideline recommendations also contribute to this challenge. For example, the 2023 PEER Simplified Lipid Guidelines by the CFPC recommend adult lipid screening starting at age 40 for men and 50 for women, but do not address or recommend pediatric screening.^23^ This discrepancy may lead to confusion among family physicians, who may prioritize CFPC guidelines over the CCS/CPCA pediatric lipid screening recommendations, potentially resulting in missed early diagnoses of conditions like FH. Although the intervention led to an increase in adherence to screening guidelines, physicians continue to exhibit hesitancy to prescribe statins to children. At the start of the intervention, 76% of participants expressed discomfort with prescribing statins, citing concerns about long-term safety in pediatric patients, despite the abundance of evidence in this field.^17^^,^^18^^,^^24^^,^^25^ After the intervention, there was a 23% improvement in confidence regarding statin prescribing. This finding illustrates that educational initiatives can be effective in addressing misconceptions and improving adherence to evidence-based treatments.

However, physician hesitancy surrounding statins is not unique to health care providers alone.^1^^,^^3^^,^^18^^,^^24^ Families and guardians of children with FH often share similar concerns about the safety and necessity of statin therapy.^1^^,^^3^^,^^18^^,^^24^ This hesitancy is compounded by a lack of awareness about the relative benefits of early pharmacologic intervention and the critical importance of managing cholesterol levels in children with FH to prevent long-term cardiovascular complications.^17^, ^18^, ^19^^,^^24^^,^^25^ To address this issue, future educational efforts should not only focus on health care providers but also on families and caregivers. By providing clear, evidence-based information about the safety and efficacy of statins, these interventions may help alleviate concerns and foster greater enthusiasm for guideline congruent pharmacologic treatment. Addressing misconceptions about statin therapy, such as fears about side effects or concerns over long-term use, is crucial for achieving optimal adherence to treatment guidelines and improving long-term cardiovascular outcomes for children with FH.

One of the strengths of our CME intervention was its interactive design, incorporating case-based learning (CBL), polling, and live question-and-answer sessions. CBL, a favored educational modality in medical schools across North America, including the majority of Canadian medical schools, proved to be more effective in engaging participants and facilitating knowledge retention compared with traditional didactic methods.^20^^,^^26^^,^^27^ Studies have shown that CBL fosters deeper understanding by encouraging active problem-solving, which is critical when applying new clinical knowledge to real-world cases.^20^^,^^26^, ^27^, ^28^, ^29^, ^30^

Feedback from participants highlighted several key areas for improvement, which have already informed iterative revisions to the course. Initial suggestions led to the simplification of the registration process and the expansion of content on statin use, pediatric prescribing, safety, and dose adjustments. Participants also expressed interest in incorporating more clinical cases to further support practical application. In addition, physicians recommended increasing access to allied health professionals, which could enhance team-based decision-making, whereas others suggested integrating the course into undergraduate medical education to ensure earlier exposure.

Given the success of our CME intervention, there is a clear opportunity to expand these educational initiatives to a broader range of health care providers. This includes not only pediatricians and family physicians, but also medical residents, nurse practitioners, pharmacists, and dietitians, all of whom play an essential role in managing pediatric dyslipidemias. These professionals could benefit from similar interventions that would enhance their knowledge and improve collaborative care in the management of FH. Expanding the intervention to include these groups could significantly enhance the reach and impact of the initiative. In addition, integrating these educational programs into undergraduate medical curricula would ensure that future physicians are better equipped to manage pediatric dyslipidemias from the outset of their careers. This could be particularly beneficial in addressing knowledge gaps and encouraging a more collaborative, multidisciplinary approach to managing FH.

Looking forward, it will be important to evaluate the long-term effectiveness of this CME intervention by tracking population-level data on FH screening, diagnosis, and treatment. Longitudinal studies will help assess whether the observed postintervention improvements in physician behavior led to measurable gains in patient care, including higher rates of screening and diagnosis, as well as increased statin prescriptions for children with genetic dyslipidemias. Despite the modest number of practitioners included in this study, the screening rates are so low that we hypothesize that if these practitioners sustain their intention to change practice, there may be appreciable changes in rates of screening and diagnosis. Furthermore, expanding the scope of this initiative requires continued effort from experts in the field. Regular CME offerings, periodic updates to content, and targeted outreach to underserved communities, especially rural and Indigenous populations, will be important to change care. Engaging with local health care providers and community stakeholders will also be essential in addressing the specific barriers to care faced by these populations. In addition, increasing family involvement in the screening and treatment process is crucial for enhancing adherence to treatment plans.^1^^,^^3^^,^^31^ As FH is a genetic disorder, increasing awareness among family members can facilitate early diagnosis and encourage broader participation in screening programs.^1^^,^^3^^,^^6^^,^^32^

### Limitations

The quasiexperimental, self-report nature of this study introduced limitations, including a lack of randomization that may have led to selection bias among participants. In addition, the absence of a control group limited comparisons to pre- and postintervention assessments within the same cohort. Self-report data further posed challenges due to biases such as social desirability, subjectivity, and over- or underestimation of behaviors, potentially affecting the reliability of the findings. Despite the accreditation of CME materials for both pediatricians and family physicians, which helped to attract participants, recruiting participants for data collection remained challenging, limiting our sample size to roughly one-third of all attendees. With only 4 family physicians participating across 3 presentation events, we could not make comparisons between family physicians and specialists. However, our participation and retention rates are consistent with those reported in comparable pre-/post-KT studies, which typically observe follow-up response rates between 10% and 30%.^33^, ^34^, ^35^, ^36^ Factors contributing to attrition may include limited time, competing clinical responsibilities, and the lack of follow-up incentives, as CME credit was awarded only at the initial session. Future efforts may benefit from linking credit to follow-up participation, offering additional incentives such as access to exclusive resources or peer networking opportunities, or using shorter, more frequent follow-up assessments to encourage sustained engagement.

With the current shortage and overburdening of primary care providers, family physicians often lack the time to participate in CME events like ours. Future studies should also explore integrating this course into the medical curriculum for residents and medical students, ensuring that these lessons are delivered early in training. This approach would eliminate the need to compete with the demanding schedules of primary care settings and administrative responsibilities. Although the KT intervention was well designed and received iterative improvements between presentation sessions, the delivery venues may have influenced its effectiveness and reach. For instance, the BC Pediatric Society session was tailored to their physician members, primarily pediatricians, whereas the RCCbc session targeted an audience of rural providers, creating variations in audience composition and potentially influencing the intervention’s outcomes.

## Conclusions

Our CME course led to significant self-reported guideline-congruent practice changes and improvements in physician confidence in managing pediatric dyslipidemias, including screening, diagnosis, and treatment, 1 month after the intervention. Expanding the reach of this KT intervention by including medical students, residents, and nurse practitioners may further support the identification and management of pediatric dyslipidemias.
