# Supplementary material for: Developing a Knowledge Translation Intervention to Improve the Detection and Management of Pediatric Dyslipidemias in British Columbia
Source: CJC Pediatr Congenit Heart Dis. 2025 May 27;4(5):283–94. doi: 10.1016/j.cjcpc.2025.05.003 (PMC12835974; doi:10.1016/j.cjcpc.2025.05.003)
Supplement: Supplemental Materials [file mmc1.docx]

**Supplemental Materials**

**Supplemental Appendix S1: Intervention Materials**

At baseline, how familiar are you with the content of the Canadian Cardiovascular Society’s 2022 clinical practice guidelines for pediatric dyslipidemias?

| Not at all familiar | Somewhat unfamiliar | Neutral | Somewhat familiar | Very familiar |
| --- | --- | --- | --- | --- |

**Patient Scenario #1- Screening**

Dania is a healthy 10-year-old female patient in your office for developmental assessment. You find their development to be normal. How likely would it be for you to order a full lipid profile for Dania?

| Very unlikely | Unlikely | Neutral | Likely | Very likely |
| --- | --- | --- | --- | --- |

What factors would make you more inclined to order a full lipid profile for Dania (select all that apply)?:

| Overweight/obese |
| --- |
| At-risk race/ethnicity |
| Chronic cardiometabolic condition (e.g. hypertension, diabetes) |
| Family history of early-onset high cholesterol |
| Family history of diabetes |
| First-degree relative with premature heart attack or stroke (men <55 years old or women <65 years old) |
| None |
| Other: (open text) |


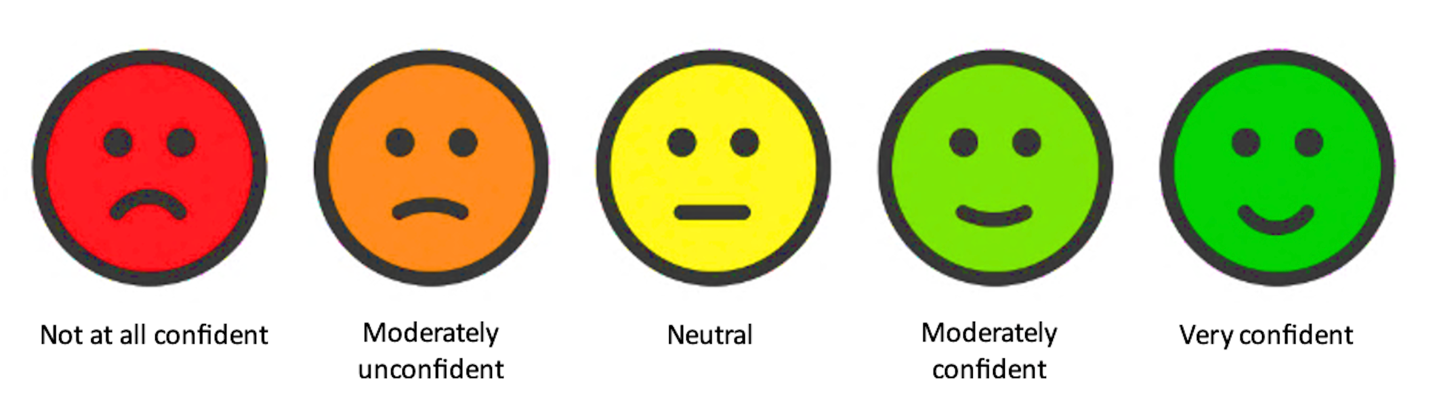
How confident were you with your selection for the previous patient, Dania? (click an icon)

**Patient Scenario #2- Diagnosis**

Results have returned from the fasting lipid profile you ordered for your 11-year-old patient Mimi. She has an LDL-C level of 5.2 mmol/L (Normal range <2.8 mmol/L). This result is consistent with a previous test ordered 3 months ago despite dietary and lifestyle changes. She does not have any other systemic disease but has a positive family history of Familial Hypercholesterolemia.

Please indicate how you would proceed from the following strategies (select all that apply):

| Repeat cholesterol test in 3 months without other changes |
| --- |
| Provide ongoing dietary and exercise counselling |
| Start statin |
| Refer to dietician |
| Offer genetic testing |
| Assess family history |
| Conduct physical examination |
| Refer to lipid specialist |
| Diagnose Mimi with Familial Hypercholesterolemia |
| None |
| Other: (open text) |

Is there another strategy you would proceed with which was NOT already presented in the previous question? (open text)


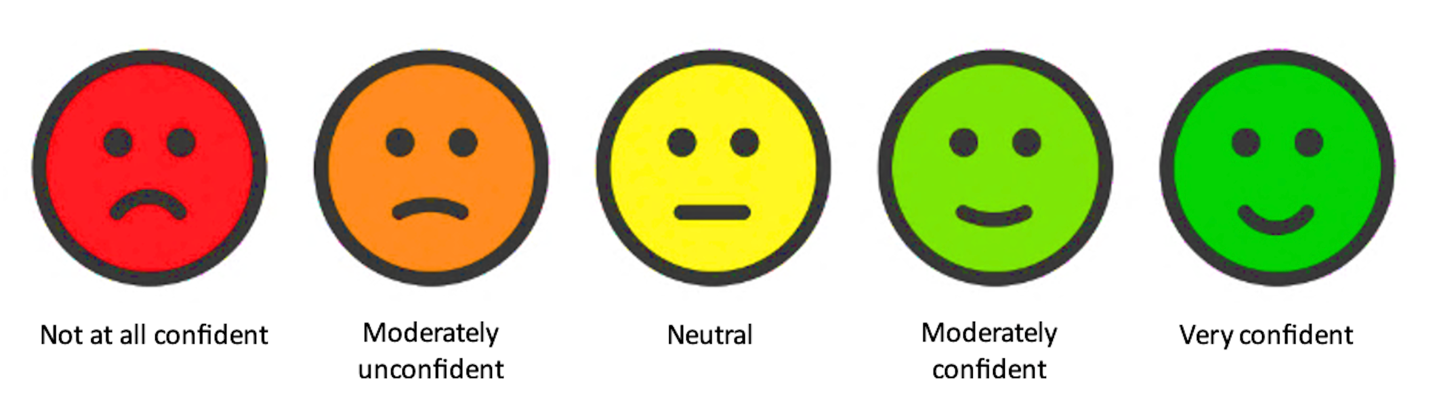
How confident are you with your selection for the previous patient, Mimi? (click an icon)

**Patient Scenario #3- Treatment**

You have diagnosed Easton, a 9-year-old patient at your office, with Familial Hypercholesterolemia (FH) based on repeated lipid profiles and a positive family history.

Please select how you would proceed with treatment at this time from the following strategies (select all that apply):

| Provide ongoing dietary and physical activity counselling |
| --- |
| Recommend cascade screening for dyslipidemias amongst Easton’s first-degree relatives |
| Start statin |
| Refer to dietician |
| Refer to lipid specialist |
| None |
| Other: (open text) |


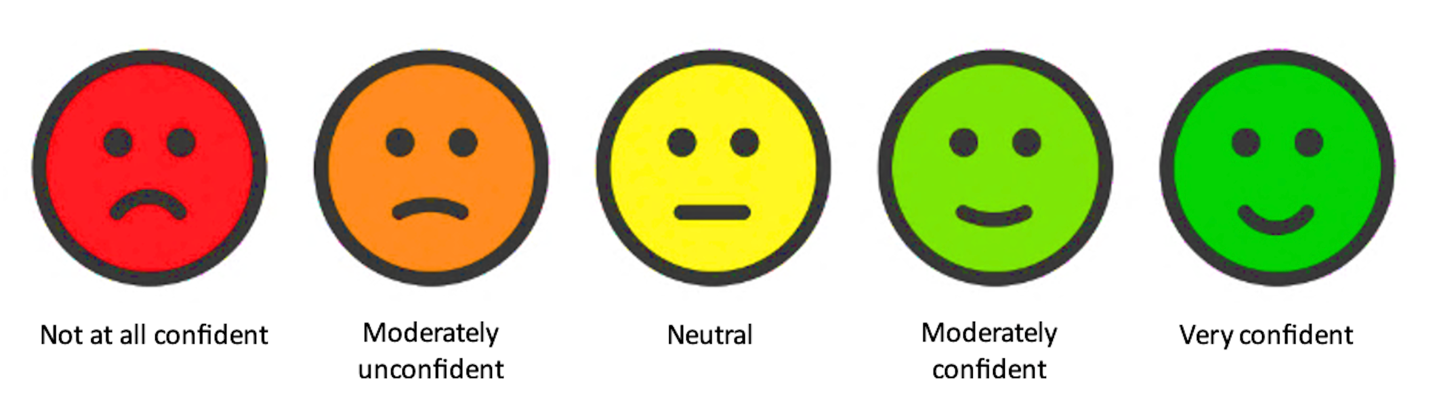
How confident are you with your selection for the previous patient, Easton? (click an icon)

**Reflection Questions**

Based on today’s module on the Canadian Cardiovascular Society’s 2022 clinical practice update for pediatric dyslipidemias, please rank to what extent you agree or disagree with each of the following statements:

I learned something new about screening and management of pediatric dyslipidemias.

| Strongly disagree | Disagree | Neutral | Agree | Strongly agree |
| --- | --- | --- | --- | --- |

What was your key takeaway? (open text)

I believe this module was an effective presentation of the 2022 CCS clinical practice guidelines for pediatric dyslipidemias.

| Strongly disagree | Disagree | Neutral | Agree | Strongly agree |
| --- | --- | --- | --- | --- |

If there is another way you would have preferred to learn about clinical practice guidelines for pediatric dyslipidemias, what is it? (open text)

What did you like about this module? (open text)

In your opinion, how could this module be improved? (open text)

After my participation in today’s module, I feel more confident managing pediatric dyslipidemias in my own practice going forward.

| Strongly disagree | Disagree | Neutral | Agree | Strongly agree |
| --- | --- | --- | --- | --- |

Following my participation in this module, I have intentions to change my current practices regarding pediatric lipid screening and/or management.

| Strongly disagree | Disagree | Neutral | Agree | Strongly agree |
| --- | --- | --- | --- | --- |

Please elaborate on what changes you intend to make to your practice, if any, and why/why not? (open text)

How familiar are you with the CCS guideline recommendations after your participation in this module?

| Not at all familiar | Somewhat unfamiliar | Neutral | Somewhat familiar | Very familiar |
| --- | --- | --- | --- | --- |

Overall, how satisfied are you with the CPD module on pediatric dyslipidemias presented by members of the Children’s Heart Centre at BC Children’s Hospital?

| Very dissatisfied | Dissatisfied | Neutral | Satisfied | Very satisfied |
| --- | --- | --- | --- | --- |

Please elaborate (open text):

**One-month Follow-up**

How familiar are you with the CCS guideline recommendations for the detection and management of primary pediatric dyslipidemias?

| Not at all familiar | Somewhat unfamiliar | Neutral | Somewhat familiar | Very familiar |
| --- | --- | --- | --- | --- |

**Patient Scenario #1- Screening**

James is a healthy 10-year-old male patient in your office for developmental assessment. You find their development to be normal. How likely would it be for you to order a full lipid profile for James?

| Very unlikely | Unlikely | Neutral | Likely | Very likely |
| --- | --- | --- | --- | --- |

What factors would make you more inclined to order a full lipid profile for James (select all that apply)?:

| Overweight/obese |
| --- |
| At-risk race/ethnicity |
| Chronic cardiometabolic condition (e.g. hypertension, diabetes) |
| Family history of early-onset high cholesterol |
| Family history of diabetes |
| First-degree relative with premature heart attack or stroke (men <55 years old or women <65 years old) |
| None |
| Other : (open text) |


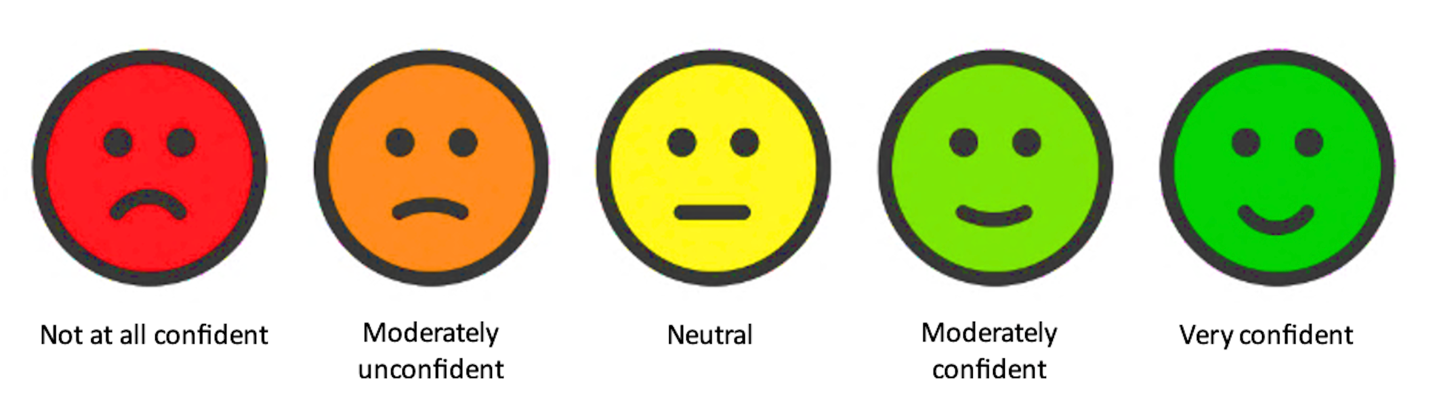
How confident were you with your selection for the previous patient, James? (click an icon)

**Patient Scenario #2- Diagnosis**

Results have returned from the fasting lipid profile you ordered for your 14-year-old patient Nicole. She has an LDL-C level of 5.2 mmol/L (Normal range <2.8 mmol/L). This result is consistent with a previous test ordered 3 months ago despite dietary and lifestyle changes. She does not have any other systemic disease but has a positive family history of Familial Hypercholesterolemia.

Please indicate how you would proceed from the following strategies (select all that apply):

| Repeat cholesterol test in 3 months without other changes |
| --- |
| Provide ongoing dietary and exercise counselling |
| Start statin |
| Refer to dietician |
| Offer genetic testing |
| Assess family history |
| Conduct physical examination |
| Refer to lipid specialist |
| Diagnose Nicole with Familial Hypercholesterolemia |
| None |
| Other: (open text) |

Is there another strategy you would proceed with which was NOT already presented in the previous question? (open text)


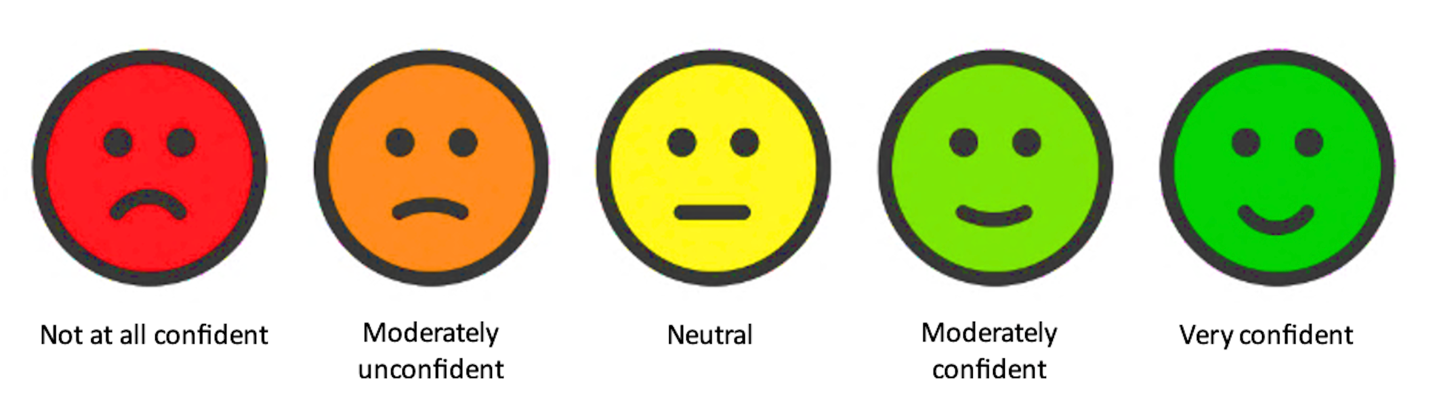


How confident are you with your selection for the previous patient, Nicole? (click an icon)

**Patient Scenario #3- Treatment**

You have diagnosed Bianca, a 9-year-old patient at your office, with Familial Hypercholesterolemia (FH) based on repeated lipid profiles and a positive family history.

Please select how you would proceed with treatment at this time from the following strategies (select all that apply):

| Provide ongoing dietary and physical activity counselling |
| --- |
| Recommend cascade screening for dyslipidemias amongst Bianca’s first-degree relatives |
| Start statin |
| Refer to dietician |
| Refer to lipid specialist |
| None |
| Other: (open text) |


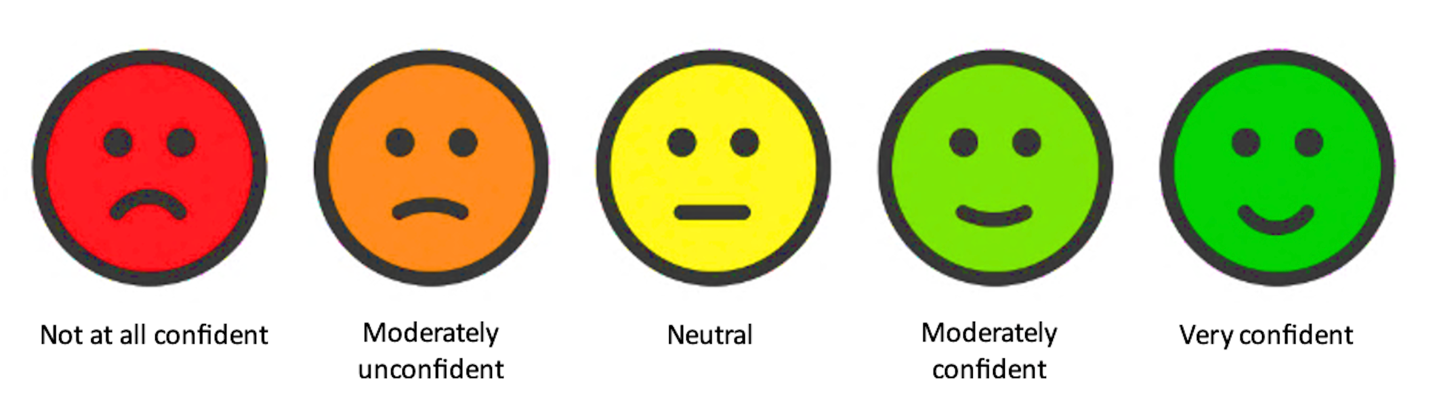
How confident are you with your selection for the previous patient, Bianca?

**One-month Follow-Up Reflection Questions**

Please rank to what extent you agree with each of the following statements:

Since my participation in the module on pediatric dyslipidemias presented by the Heart Centre at BC Children’s Hospital, I have felt more confident managing pediatric dyslipidemias in my own practice.

| Strongly disagree | Disagree | Neutral | Agree | Strongly agree |
| --- | --- | --- | --- | --- |

Following my participation in the CPD module on pediatric dyslipidemias presented by the Children’s Heart Centre at BC Children’s Hospital, I have made changes my current practices regarding pediatric lipid screening and/or management.

| Strongly disagree | Disagree | Neutral | Agree | Strongly agree |
| --- | --- | --- | --- | --- |

Please elaborate on what changes you made to your practice, if any, and why/why not? (open text)

Overall, how satisfied are you with the CPD module on pediatric dyslipidemias presented by members of the Children’s Heart Centre at BC Children’s Hospital?

| Very dissatisfied | Dissatisfied | Neutral | Satisfied | Very satisfied |
| --- | --- | --- | --- | --- |

Please elaborate (open text):

**(Optional)** Please provide an email address to be contacted about study results.

Preferred email for future correspondence:

**Supplemental Appendix S2: Intervention Demographics Survey**

**Supplemental Appendix S3: Post-Intervention Key Point Summary**


**Supplemental Figure S1.** Comparison of acceptable, borderline, and abnormal pediatric lipid thresholds outlined by the American Academy of Pediatrics and the Canadian Cardiovascular Society clinical practice guidelines, shared with participants during and after the intervention.

**Supplemental Figure S2.** Simplified clinical decision-making flowchart for the screening, diagnosis, and management of pediatric dyslipidemias, distributed to participants following the intervention.
